# Iliotibial Band Autograft Provides the Fastest Recovery of Knee Extensor Mechanism Function in Pediatric Anterior Cruciate Ligament Reconstruction

**DOI:** 10.3390/ijerph18147492

**Published:** 2021-07-14

**Authors:** Tishya L. Wren, Veronica Beltran, Mia J. Katzel, Adriana S. Conrad-Forrest, Curtis D. VandenBerg

**Affiliations:** 1 Children’s Orthopaedic Center, Children’s Hospital Los Angeles, Los Angeles, CA 90027, USA; vbeltran@chla.usc.edu (V.B.); mkatzel@chla.usc.edu (M.J.K.); aconradforrest@chla.usc.edu (A.S.C.-F.); cvandenberg@chla.usc.edu (C.D.V.); 2 Department of Orthopaedic Surgery, Keck School of Medicine, University of Southern California, Los Angeles, CA 90033, USA

**Keywords:** anterior cruciate ligament autograft, biomechanics, cutting, drop jump, pediatric athlete, knee injury, surgical decision making

## Abstract

Iliotibial band autograft is an increasingly popular option for pediatric anterior cruciate ligament reconstruction (ACLR). The purpose of this study was to compare recovery of knee extensor mechanism function among pediatric patients who underwent ACLR using iliotibial band (IT), hamstring tendon (HT), quadriceps tendon (QT), and patellar tendon (PT) autografts. One hundred forty-five pediatric athletes (76 female; age 15.0, range 7–21 years) with recent (3–18 months) unilateral ACLR performed drop-jump landing and 45° cutting with 3D motion capture. Knee extensor mechanism function (maximum knee flexion angle, maximum internal knee extensor moment, energy absorption at knee) during the loading phase (foot contact to peak knee flexion) was compared among graft types (20 IT, 29 HT, 39 QT, 57 PT) and sides (ACLR or contralateral) using linear mixed models with sex, age, and time since surgery as covariates. Overall, knee flexion was significantly lower on the operated vs. contralateral side for HT, QT, and PT during both tasks (*p* < 0.03). All graft types exhibited lower knee extensor moments and energy absorption on the operated side during both movements (*p* ≤ 0.001). Kinetic asymmetry was significantly lower for IT compared with QT and PT during both movements (*p* ≤ 0.005), and similar patterns were observed for HT vs. QT and PT (*p* ≤ 0.07). Asymmetry was similar between IT and HT and between QT and PT. This study found that knee extensor mechanism function recovers fastest in pediatric ACLR patients with IT autografts, followed by HT, in comparison to QT and PT, suggesting that IT is a viable option for returning young athletes to play after ACLR.

## 1. Introduction

Anterior cruciate ligament reconstruction (ACLR) is increasingly common in pediatric athletes [1,2]. Treatment is individualized considering a patient’s age, current status, and future goals. The main objective of ACLR surgery is to restore stability and integrity of the knee joint and function of the limb [3]. Surgical decisions including graft selection, tunnel placement, graft tension, and fixation technique are made to optimize surgical outcomes [4]. These surgical decisions aim to restore pre-injury function, avoid growth disturbances, minimize the risk of future non-contact injury (ipsilateral re-tear or subsequent contralateral ACL tear), and maximize patient satisfaction. One important decision in ACLR is selection of graft type. While multiple studies have shown clear benefits of autograft over allograft for ACLR in young athletes [3,5], disagreement remains regarding the optimal autograft choice. Graft selection for pediatric ACLR is typically based on surgeon preference, physical maturity status of the patient, and post-surgical goals of the athlete.

Innovations in surgical technique, especially for younger, highly active patients, have improved outcomes and made ACLR the preferred treatment option even at younger ages [6]. ACLR in skeletally immature patients requires extra care due to the risk of growth disturbance or development of angular deformity if open growth plates are damaged [6,7,8]. Many ACLR surgical techniques have been described which allow for continued growth in patients with open physes, including techniques that avoid crossing the growth plate [6,9]. These include ACLR using iliotibial (IT) band autograft which has produced excellent functional outcomes in skeletally immature, prepubescent children [6,10]. Technical advantages of the IT band autograft ACLR technique include avoidance of any tunnel reaming, along with this technique resulting in combined intraarticular and extraarticular ligamentous reconstruction [10,11,12]. Despite these improvements in surgical technique, young and highly active patients have the highest risk for ACL re-injury once they are released back to sport [13] with some studies reporting re-injury rates as high as 30% in pediatric athletes [14]. Evidence has shown that adolescent and young adult athletes’ biomechanical movement patterns can predict risk for non-contact ACL injury and re-injury. Hewett et al. showed that ACL injury risk decreased when athletes’ biomechanical deficits were addressed [15]. Research by Paterno et al. [16] and Leppanen et al. [17] has also shown that sagittal plane kinematics and kinetics can predict ACL re-injury.

One important prerequisite for returning to activity after ACLR is recovery of knee extensor strength and dynamic function [3]. The progression of this recovery is often evaluated by assessing symmetry between the reconstructed and contralateral limb [3,18,19] although this comparison has limitations due to potential changes in performance of the contralateral limb due to reduction of activity [18,20,21]. The timeline for recovery can vary greatly among individuals and may be influenced by specifics of the surgery such as graft type and fixation technique [4]. Young athletes who do not have 90% quadriceps strength symmetry at the time of return to play demonstrate persistent decreased knee function and functional recovery one year later [19].

While the current literature clearly demonstrates the importance of surgical management and biomechanics in predicting re-injury rates in pediatric athletes after primary ACLR [4,22], limited data are available to determine how graft selection affects young athletes’ biomechanics and rehabilitation trajectories. We have previously shown that pediatric patients reconstructed with patellar tendon or quadriceps tendon autografts demonstrate greater biomechanical deficiencies during the rehabilitation phase than those reconstructed with hamstring tendon autografts [23]. This study expands on that previous research by including patients with iliotibial band autografts, focusing on recovery of function of the knee extensor mechanism. Specifically, the current study compares sagittal plane knee biomechanics among pediatric patients with different types of ACLR autografts including iliotibial band (IT), hamstring tendon (HT), quadriceps tendon (QT), and patellar tendon (PT). We hypothesized that knee extensor function would recover faster for graft types that did not disrupt the knee extensor mechanism (i.e., IT and HT vs. PT and QT).

## 2. Materials and Methods

This study examined retrospective clinical data from pediatric athletes ages 7–21 years who had undergone sports biomechanical testing in our motion analysis laboratory following recent unilateral ACLR with surgery dates between June 2014 and August 2019. All ACL injuries were primary, i.e., patients with prior ACL injury were excluded. Patients with concomitant injury to other knee ligaments were also excluded, but meniscus injuries were allowed (see Table 1). Patients with meniscus injuries were included because these injuries commonly accompany ACL injury, and excluding them would make the sample less representative of the population with ACLR. Stratified analysis was performed to separately examine patients with and without concomitant meniscus procedures. The choice of autograft type and ACL reconstruction technique was individualized for each patient depending on skeletal age and activity goals. All reconstructions utilized IT, HT, QT, or PT autografts (Table 1), and particular attention was paid to minimizing risk of injury to open physes while optimizing restoration of normal knee biomechanics. Surgical techniques included IT band physeal-avoiding ACLR, hamstring or quadriceps tendon soft-tissue transphyseal or partial all-epiphyseal and transphyseal ACLR with suspensory fixation, and traditional bone–patella tendon–bone autograft ACLR with interference screw fixation. The surgeons’ preferred treatment algorithm included a recommendation for physis-avoiding IT band ACLR for all prepubescent patients. Pubescent or post-pubescent patients with greater than one year of growth remaining underwent all-epiphyseal femur and transphyseal tibia ACLR with QT or HT. Patients with less than one year of growth remaining underwent transphyseal reconstruction with QT or HT. Skeletally mature patients underwent traditional ACLR using PT, QT, or HT. Rehabilitation focused on range of motion, strengthening, and restoration of gait initially and progressed to impact activities, plyometrics, and sport-specific training with emphasis on neuromuscular control and was individualized by physical therapists in the community. All participants and the parents of minors either provided written informed assent and consent for their data to be used in research, or data were accessed retrospectively under a waiver of consent granted by our hospital’s institutional review board.

Biomechanical testing was performed 3–18 months post-surgery to guide rehabilitation and/or to assess return to sport readiness. Some participants had more than one biomechanical test at different timepoints, and all were included in the analysis as repeated measures. Generally, earlier tests were performed to identify biomechanical deficits to work on during rehabilitation, while later tests were performed to evaluate return to play readiness. Biomechanical testing included vertical drop jump (DJ) and side-step cutting tasks along with other tasks not examined in the current study. For the vertical drop jump, participants were instructed to drop off a 41 cm box, land with both feet on separate force plates, and then immediately jump straight up as high as possible, landing back on the same force plates to keep the jump vertical. For cutting, participants were instructed to run forward at their maximum speed, plant on the force plate (right foot if cutting to the left or vice versa), and cut towards the contralateral side at a 45° angle along a guideline taped on the floor. At the start of the session, each participant warmed up similar to a typical physical therapy session, including functional warm-ups, followed by about 60 s of walking and a minimum of 90 s of jogging on a treadmill. They performed 2–3 practice trials for each task prior to data collection for that task. Then, data were collected for three trials per limb, and all useable data for each side were averaged for analysis.

Data were collected using a 10-camera motion capture system at 120 Hz (Nexus 2, Vicon Motion Systems Ltd., Oxford, UK) and 4 analog force plates at 2400 Hz (AMTI OR6–5, Advanced Medical Terminology, Inc., Watertown, MA, USA). Markers were taped over anatomic landmarks following a modified Plug-in-Gait (Vicon Motion Systems Ltd., Oxford, UK) marker set with anterior thigh clusters, two tibial crest markers, and a marker on the shoe above the 5th metatarsal head [24]. Marker trajectories were filtered using a Woltring filter with a mean squared error of 10 mm^2^, and force plate data were filtered using a 16 Hz Butterworth filter. Kinematics and kinetics of the trunk and lower extremities were calculated using a 6-degree-of-freedom model [24] in Visual3D (C-Motion, Inc., Germantown, MD, USA) and were evaluated from initial contact to peak knee flexion representing the loading phase of the DJ landing or cut when injury is most likely to occur [25,26].

To assess dynamic function of the knee extensor mechanism, we analyzed maximum knee flexion angles, maximum internal knee extensor moments, and energy absorption at the knee (integral of the power curve) during the landing phase of each movement as defined above. These outcome measures were compared among graft types (IT, HT, QT, or PT) and sides (ACLR or contralateral) using linear mixed models with sex, age, and time since surgery as covariates. Graft type, side, and covariates were included as fixed effects, and a random intercept was included for participant to account for the repeated measures. This analysis was repeated for subgroups stratified by whether a meniscus procedure was done (isolated ACLR or ACLR with concomitant meniscus repair, debridement, or menisectomy) and also for only patients under 16 years of age and visits at least 6 months post-surgery. All analyses were performed in Stata (version 14, StataCorp LLC, College Station, TX, USA) with a significance level of 0.05.

## 3. Results

The study sample included 145 pediatric athletes (76 female; mean age at surgery 15.0, SD 2.2, range 7–21 years) including 20 IT, 29 HT, 39 QT, and 57 PT (Table 1). One hundred sixteen participants underwent a single test, 28 had two tests, and 1 had three tests, yielding 175 total tests (mean 7.4, SD 2.7, range 3–18 months post-surgery). Sex (*p* = 0.03) and age (*p* < 0.001) differed significantly among graft types with the IT group being younger with a higher percentage of males and the PT group being older than the other groups (Table 1). These differences were accounted for in the analyses below by including sex, age, and time since surgery as covariates.

**Figure 1:**
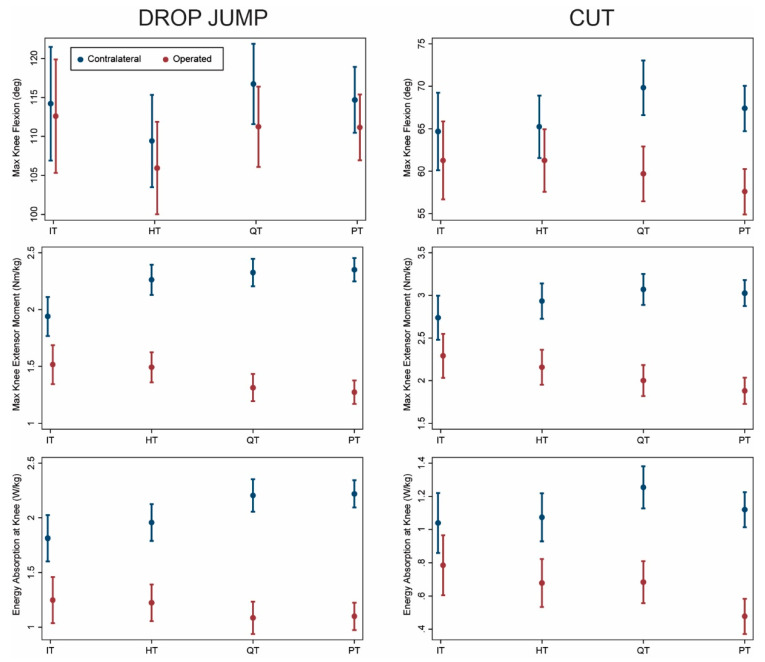
Comparison of operated (red) and contralateral (blue) limbs by graft type (model predicted mean and 95% confidence interval) for complete sample. IT: iliotibial band; HT: hamstring tendon; QT: quadriceps tendon; PT: patellar tendon.

Comparing graft types after adjusting for covariates in the overall sample, dynamic knee extensor function of the operated limb was greatest in the IT group followed by the HT group as discussed below. Knee flexion angle was significantly lower on the operated vs. contralateral side for HT, QT, and PT during both drop jump (*p* < 0.03, Table 2) and cutting (*p* < 0.007, Table 3). All graft types exhibited lower knee extensor moments and energy absorption on the operated side during both movements (*p* ≤ 0.001). This asymmetry was most pronounced for QT and PT and least pronounced for IT (Figure 1). Loading on the operated limb decreased in order from IT to HT to QT and PT, while loading on the contralateral limb increased similarly. Asymmetry of kinetics (difference between sides) was significantly lower for IT compared with both QT and PT during both movements (*p* ≤ 0.005). Similar patterns were observed for HT but were less pronounced and not always statistically significant (*p* ≤ 0.07). Asymmetry differed between IT and HT only for knee extensor moments during both tasks (*p* ≤ 0.05), and no differences in asymmetry were observed between QT and PT (*p* ≥ 0.16).

### 3.1. Isolated ACL Reconstruction

**Figure 2:**
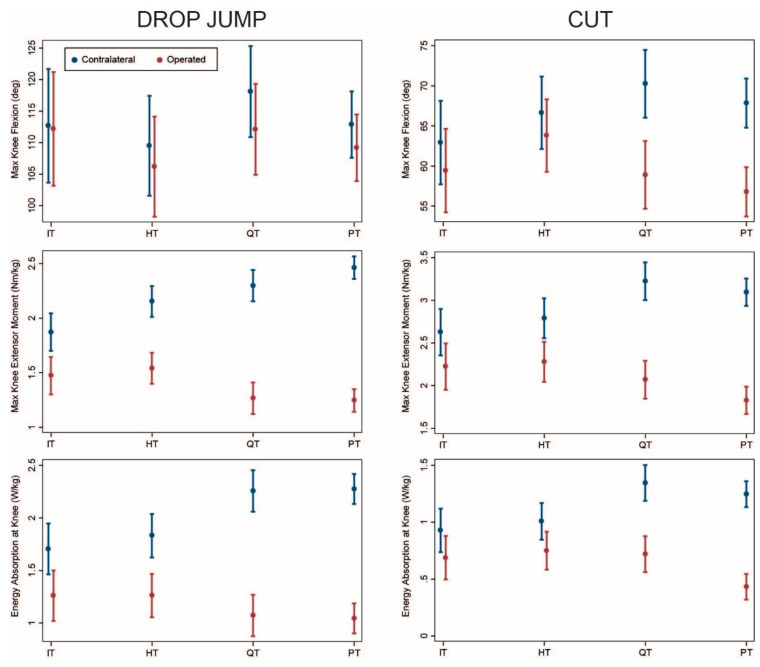
Comparison of operated (red) and contralateral (blue) limbs by graft type (model predicted mean and 95% confidence interval) for patients with isolated ACLR (no concomitant meniscus procedures). IT: iliotibial band; HT: hamstring tendon; QT: quadriceps tendon; PT: patellar tendon.

Ninety-five patients underwent isolated ACLR without concomitant meniscus surgery (14 IT, 18 HT, 22 QT, 41 PT; Table 1). Results for this group were similar to the overall group (Figure 2). Knee flexion angle was significantly lower on the operated vs. contralateral side for HT, QT, and PT during drop jump (Table A1) and for QT and PT during cutting (Table A2). Significant asymmetry of knee moments and energy absorption was observed for all graft types, with asymmetry being greater for QT and PT compared with IT and HT during both drop jump and cutting (*p* ≤ 0.003). Asymmetry was similar between IT and HT (*p* ≥ 0.17) and between QT and PT (*p* ≥ 0.10).

### 3.2. ACL Reconstruction with Meniscus Procedure

**Figure 3:**
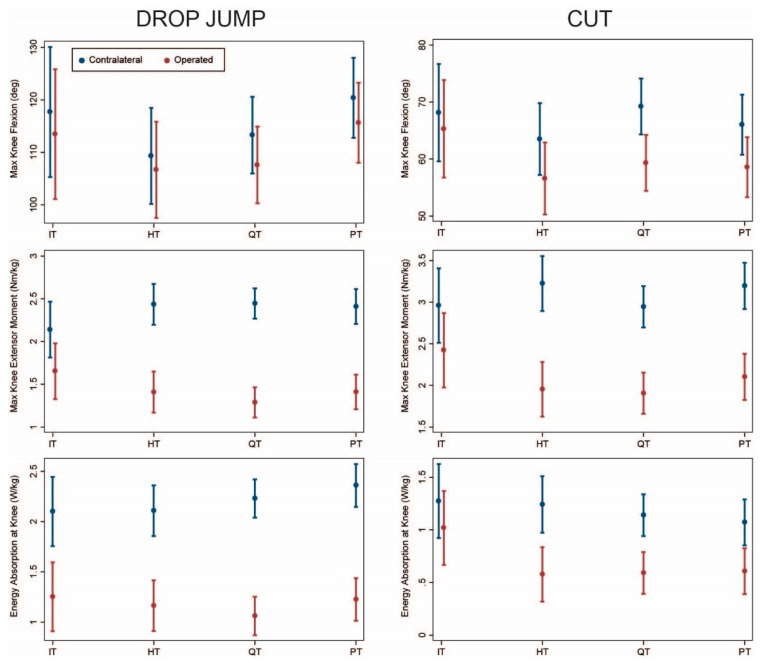
Comparison of operated (red) and contralateral (blue) limbs by graft type (model predicted mean and 95% confidence interval) for patients with concomitant meniscus procedures. IT: iliotibial band; HT: hamstring tendon; QT: quadriceps tendon; PT: patellar tendon.

Fifty patients underwent concomitant meniscus surgery (repair, debridement, or menisectomy) at the time of their ACLR (6 IT, 11 HT, 17 QT, 16 PT; Table 1). The results showed similar trends to the overall sample with the IT group demonstrating the least asymmetry (Figure 3; Table A3 and Table A4). However, uncertainty was greater in this analysis (larger standard errors and confidence intervals) because of the small sample size. The magnitude of asymmetry was generally similar to the overall analysis (within 3°, 0.2 Nm/kg, or 0.2 J/kg) except for a higher estimated asymmetry of energy absorption for IT during drop jump (−0.9 J/kg for this subgroup vs. −0.6 J/kg for the overall group) and higher estimated asymmetry of knee moments (−1.3 Nm/kg for this subgroup vs. −0.8 Nm/kg for the overall group) and energy absorption (−0.7 J/kg for this subgroup vs. −0.4 J/kg for the overall group) for HT during cutting. In most cases, there was no statistically significant difference in asymmetry among groups. 

### 3.3. Younger Patients at Least 6 Months Post-Surgery

**Figure 4:**
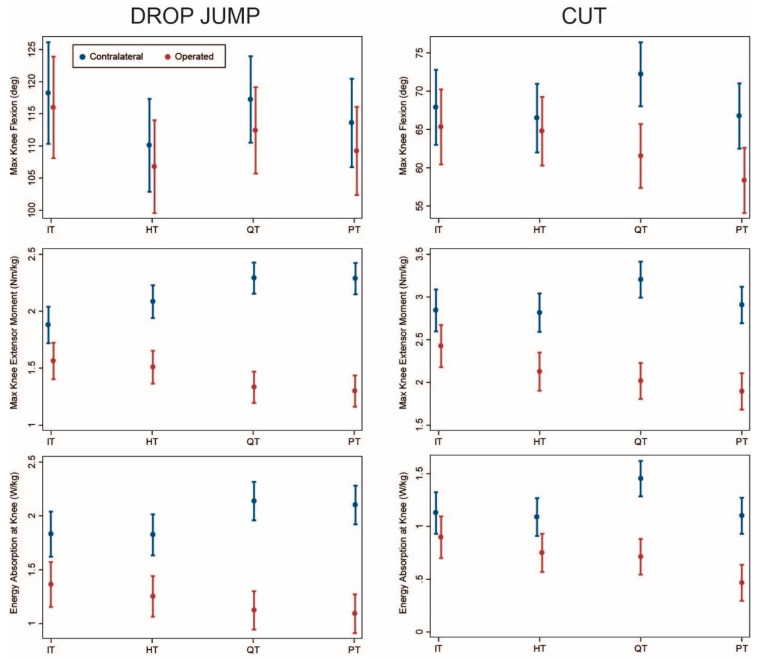
Comparison of operated (red) and contralateral (blue) limbs by graft type (model predicted mean and 95% confidence interval) for patients under 16 years of age and visits at least 6 months post-surgery. IT: iliotibial band; HT: hamstring tendon; QT: quadriceps tendon; PT: patellar tendon.

Seventy-eight patients (16 IT, 19 HT, 22 QT, 21 PT) who were under 16 years of age at the time of surgery underwent 84 assessments (17 IT, 21 HT, 23 QT, 23 PT) at least 6 months post-surgery. Again, the results for this subgroup were similar to the overall sample with the least asymmetry for IT followed by HT (Figure 4). Knee moments and energy absorption were significantly lower on the operated side compared with the contralateral side for all graft types during both drop jump (Table A5) and cutting (Table A6). This kinetic asymmetry was greater for QT and PT compared with IT and HT during both movements (*p* ≤ 0.05). Asymmetry did not differ significantly between IT and HT (*p* ≥ 0.08) or between QT and PT (*p* ≥ 0.27). 

## 4. Discussion

This study compared the recovery of knee extensor mechanism function among young athletes with different types of ACLR autografts. In the rehabilitation and return to sport timeframe following ACLR, young athletes with IT band autografts exhibited the greatest engagement of the knee extensors during dynamic loading among all autograft types studied. This was evidenced by both higher loading of the reconstructed knee and lower loading of the contralateral knee. Patients with HT grafts demonstrated the second-best recovery of knee extensor function. Slower recovery of the knee extensor mechanism in patients with PT and QT autografts is not surprising since graft harvest directly affects the extensor mechanism and creates the possibility of donor site morbidity. Patients and providers should be cognizant that recovery of knee extensor function may be slower in patients reconstructed with PT or QT autografts. 

However, knee extensor recovery time is not the only factor to be considered when selecting the graft type to be used in ACLR. In addition to surgeon preference, patient age and skeletal maturity are important considerations in surgical management due to the potential for growth disturbance in skeletally immature patients [3,7,27]. IT grafts are typically used for the youngest patients, who have the greatest remaining growth, and are combined with physis-avoiding techniques [7,9]. As patients mature, preferred treatment progresses to HT or QT soft tissue grafts with physis-respecting transphyseal or partial transphyseal fixation [7,27]. Skeletally mature patients or those with little growth remaining usually undergo traditional adult-type reconstruction with PT, QT, or HT grafts [3,27]. Previous studies have indicated good clinical results for IT band ACLR [6,10]. This study adds quantitative data showing faster restoration of knee kinematics and kinetics compared with other graft types.

Due to the age-related progression in surgical management, our groups differed in age as expected, with the IT group being youngest, followed by the HT group. The distribution of sex also differed among groups, with a higher percentage of males in the IT (75% male) and HT (55% male) groups. The difference in sex is likely related to the difference in age since ACL injury is more common in boys at younger ages (<12 years) and girls at older ages (≥12 years) [28]. To account for these differences, we included sex and age as covariates in our analysis. We also performed a subanalysis including only patients aged 15 years old and younger and obtained similar results to those presented for the entire sample.

ACLR using IT band autograft offers the advantage of physis avoidance for younger, skeletally immature patients [9]. The procedure is highly successful in pre-pubertal patients, achieving excellent knee stability and patient-reported outcomes an average of 6 years post-operatively [10]. In our study, the IT group exhibited slightly less asymmetry than the HT group (Figure 1, Figure 2, Figure 3 and Figure 4). The IT and HT groups differed from the QT and PT groups on most measures of kinetic asymmetry. Little difference was observed between the QT and PT groups in kinematics or kinetics.

Asymmetry reflecting offloading of the reconstructed knee was much more evident in kinetic measures compared with kinematics. In the overall sample, peak knee flexion angle only differed by more than 10° between limbs for the PT and QT groups during cutting. For the IT and HT groups during cutting and for all groups during the drop jump, asymmetry averaged only 2–6° (2–6% of mean values) which would be difficult to discern visually and might not be considered clinically significant. In contrast, moments differed by 15–50% (0.4–1.2 Nm/kg), and energy absorption differed by 20–58% (0.2–1.2 J/kg). This highlights the importance of kinetics in evaluating functional recovery and return to play readiness after ACLR.

Limitations of this study include the wide range of time since surgery and the difference in sex and age among groups. As noted above, we adjusted for these factors as covariates in our analyses, but it is possible that this did not fully account for the effects of these variables. To further examine these effects, we performed a subanalysis excluding visits less than 6 months post-surgery and excluding patients older than age 15 years. This subanalysis produced similar results as for the whole group. We also performed subanalyses stratifying patients based on whether or not they underwent concomitant meniscus procedures. Again, similar results were obtained although there was greater uncertainty in the results for the subgroup with meniscus procedures because of the smaller sample size. Meniscus repair can affect the rehabilitation timeline during the first 6–8 weeks post-surgery, but patients are expected to recover similarly to those with isolated ACLR by 3–4 months post-surgery which is the earliest the patients in our study were assessed. Meniscus debridement and menisectomy do not alter the rehabilitation protocol. This was also a retrospective study in which treatment was not randomized, and the sample size is limited, especially for the IT group. While we believe the sample size is adequate for the current analysis, it was not large enough to allow for subanalysis stratified by sex. The rehabilitation programs were not standardized and reflected typical physical therapy provided in the community. Additionally, the study sample derived from a single pediatric hospital with two board-certified pediatric orthopedic surgeons performing almost all of the surgeries. Larger multi-center studies would be needed to confirm the generalizability of our results.

## 5. Conclusions

In conclusion, knee extensor mechanism function appears to recover fastest in pediatric ACLR patients reconstructed with IT autografts, followed by HT autografts. Knee extensor function recovers more slowly in patients reconstructed with PT and QT autografts. While these graft types are typically used in different age groups, our results suggest that IT band autografts may be a good option for returning young athletes to play. Thus, our findings support the use of IT bands as a viable autograft option in young athletes undergoing ACLR.

## Figures and Tables

**Table 1:** Participant characteristics.

	IT (*n* = 20 Subjects)	HT (*n* = 29 Subjects)	QT (*n* = 39 Subjects)	PT (*n* = 57 Subjects)	*p*-Value	All (*n* = 145 Subjects)
Sex					0.03	
Female	5 (25%)	13 (45%)	23 (59%)	35 (61%)		876 (52%)
Male	15 (75%)	16 (55%)	16 (41%)	22 (39%)		69 (48%)
Age at surgery (years)	11.3 (1.6)	14.5 (1.0)	15.0 (1.4)	16.4 (1.6)	<0.001	15.0 (2.2)
Height (cm)	151.1 (13.3)	169.2 (9.8)	165.9 (9.2)	166.9 (10.7)	<0.001	164.9 (11.9)
Body mass (kg)	45.8 (12.2)	74.0 (25.3)	64.2 (14.2)	69.1 (15.8)	<0.001	65.5 (19.2)
BMI (kg/m^2^)	19.9 (4.5)	25.4 (6.7)	23.2 (3.5)	24.7 (5.1)	0.001	23.8 (5.3)
Meniscus procedure					0.09	
None	14 (70%)	18 (62%)	22 (56%)	41 (72%)		95 (66%)
Debridement	0	0	2 (5%)	2 (4%)		4 (3%)
Repair	6 (30%)	9 (31%)	10 (26%)	8 (14%)		33 (23%)
Menisectomy	0	2 (7%)	5 (13%)	6 (11%)		13 (9%)
Sports						
Baseball/Softball	4 (20%)	5 (17%)	3 (8%)	3 (5%)	0.15	15 (10%)
Basketball	9 (45%)	6 (21%)	11 (28%)	15 (26%)	0.30	41 (28%)
Dance/Cheer/Gymnastics	1 (5%)	2 (7%)	3 (8%)	5 (9%)	0.96	11 (8%)
Football	7 (35%)	8 (28%)	7 (18%)	7 (12%)	0.11	29 (20%)
Soccer	8 (40%)	10 (34%)	16 (41%)	22 (39%)	0.96	56 (39%)
Track/Cross-country	0	0	4 (13%)	9 (16%)	0.04	13 (10%)
Volleyball	2 (10%)	5 (17%)	4 (10%)	6 (11%)	0.79	17 (12%)
Other	8 (40%)	6 (21%)	10 (26%)	11 (19%)	0.30	35 (24%)
Time playing each sport (h/week)	5.8 (3.1)	9.7 (6.1)	8.5 (4.3)	10.6 (7.1)	<0.001	9.1 (6.0)
	*n* = 24 visits	*n* = 38 visits	*n* = 48 visits	*n* = 65 visits		*n* = 175 visits
Time post-surgery (months)	7.8 (3.4)	7.0 (3.0)	6.8 (2.2)	7.9 (2.5)	0.09	7.4 (2.7)

Continuous variables are presented as mean (SD) and compared using ANOVA. Categorical variables are presented as *n* (%) and compared using Fisher’s exact test. IT: iliotibial band; HT: hamstring tendon; QT: quadriceps tendon; PT: patellar tendon.

**Table 2:** Comparison of sagittal knee kinematics/kinetics for operative and non-operative limbs during vertical drop jump for complete sample.

Drop Jump	Mean (SE)				*p*-Value					
All Subjects	IT (*n* = 24 Visits)	HT (*n* = 38 Visits)	QT (*n* = 48 Visits)	PT (*n* = 65 Visits)	HT vs. IT	QT vs. IT	PT vs. IT	QT vs. HT	PT vs. HT	PT vs. QT
Max Knee Angle (°)										
Non-op	114 (4)	109 (3)	116 (3)	115 (2)	0.39	0.81	0.79	0.11	0.47	0.36
Op	113 (4)	106 (3)	110 (3)	111 (2)	0.27	0.66	0.54	0.35	0.60	0.67
Asymmetry	−2 (2)	**−3 (1)**	**−6 (1)**	**−4 (1)**	0.48	0.04	0.22	0.13	0.60	0.24
Max Knee Moment (N·m/kg)										
Non-op	2.0 (0.1)	2.3 (0.1)	2.4 (0.1)	2.4 (0.05)	0.06	0.006	0.004	0.21	0.08	0.57
Op	1.5 (0.1)	1.5 (0.1)	1.3 (0.1)	1.3 (0.05)	0.33	0.004	0.005	0.007	0.004	0.83
Asymmetry	**−0.4 (0.1)**	**−0.8 (0.1)**	**−1.1 (0.1)**	**−1.2 (0.1)**	0.02	<0.001	<0.001	0.005	<0.001	0.56
Energy Absorption at Knee (J/kg)										
Non-op	1.8 (0.1)	1.9 (0.1)	2.2 (0.1)	2.3 (0.1)	0.92	0.04	0.07	0.005	0.007	1.00
Op	1.3 (0.1)	1.2 (0.1)	1.1 (0.1)	1.1 (0.1)	0.43	0.06	0.07	0.11	0.07	0.78
Asymmetry	**−0.6 (0.1)**	**−0.7 (0.1)**	**−1.2 (0.1)**	**−1.2 (0.1)**	0.35	<0.001	<0.001	<0.001	<0.001	0.79

Results are presented as model predicted mean (SE) adjusting for sex, age, and time since surgery. Bold indicates significant difference between sides (asymmetry) within each group at *p* < 0.05. IT: iliotibial band; HT: hamstring tendon; QT: quadriceps tendon; PT: patellar tendon.

**Table 3:** Comparison of sagittal knee kinematics/kinetics for operative and non-operative limbs during cutting for complete sample.

Cut	Mean (SE)				*p*-Value					
All Subjects	IT (*n* = 24 Visits)	HT (*n* = 38 Visits)	QT (*n* = 48 Visits)	PT (*n* = 65 Visits)	HT vs. IT	QT vs. IT	PT vs. IT	QT vs. HT	PT vs. HT	PT vs. QT
Max Knee Angle (°)										
Non-op	65 (2)	65 (2)	70 (2)	67 (1)	0.78	0.17	0.89	0.10	0.88	0.05
Op	61 (2)	61 (2)	59 (2)	57 (1)	0.99	0.54	0.14	0.36	0.02	0.09
Asymmetry	−3 (2)	**−4 (2)**	**−11 (1)**	**−10 (1)**	0.71	0.002	0.003	0.002	0.002	0.74
Max Knee Moment (N·m/kg)										
Non-op	2.7 (0.1)	2.9 (0.1)	3.1 (0.1)	3.1 (0.1)	0.18	0.03	0.03	0.17	0.13	0.83
Op	2.3 (0.1)	2.2 (0.1)	2.0 (0.1)	1.9 (0.1)	0.64	0.22	0.12	0.27	0.08	0.42
Asymmetry	**−0.4 (0.1)**	**−0.8 (0.1)**	**−1.1 (0.1)**	**−1.2 (0.1)**	0.05	<0.001	<0.001	0.02	0.001	0.32
Energy Absorption at Knee (J/kg)										
Non-op	1.0 (0.1)	1.1 (0.1)	1.2 (0.1)	1.2 (0.1)	0.71	0.11	0.38	0.08	0.40	0.32
Op	0.8 (0.1)	0.7 (0.1)	0.7 (0.1)	0.5 (0.1)	0.48	0.39	0.03	0.82	0.02	0.01
Asymmetry	**−0.2 (0.1)**	**−0.4 (0.1)**	**−0.6 (0.1)**	**−0.7 (0.1)**	0.25	0.005	<0.001	0.07	0.001	0.16

Results are presented as model predicted mean (SE) adjusting for sex, age, and time since surgery. Bold indicates significant difference between sides (asymmetry) within each group at *p* < 0.05; IT: iliotibial band; HT: hamstring tendon; QT: quadriceps tendon; PT: patellar tendon.

## Data Availability

Deidentified data may be made available upon request with approval of the Institutional Review Board.

## Appendix A

**Table A1:** Comparison of sagittal knee kinematics/kinetics for operative and non-operative limbs during vertical drop jump for patients with isolated ACLR (no concomitant meniscus procedure).

Drop Jump	Mean (SE)				*p*-Value					
Isolated ACLR	IT (*n* = 17 Visits)	HT (*n* = 25 Visits)	QT (*n* = 24 Visits)	PT (*n* = 47 Visits)	HT vs. IT	QT vs. IT	PT vs. IT	QT vs. HT	PT vs. HT	PT vs. QT
Max Knee Angle (°)										
Non-op	113 (5)	110 (4)	118 (4)	113 (3)	0.80	0.47	0.96	0.14	0.77	0.77
Op	112 (5)	106 (4)	112 (4)	109 (3)	0.56	0.95	0.71	0.33	0.82	0.42
Asymmetry	−0.5 (2)	**−3 (2)**	**−6 (2)**	**−4 (1)**	0.26	0.03	0.15	0.24	0.85	0.24
Max Knee Moment (N·m/kg)										
Non-op	1.9 (0.1)	2.2 (0.1)	2.3 (0.1)	2.5 (0.1)	0.04	0.002	<0.001	0.11	0.001	0.07
Op	1.5 (0.1)	1.5 (0.1)	1.3 (0.1)	1.2 (0.1)	0.61	0.20	0.21	0.01	0.008	0.92
Asymmetry	**−0.4 (0.1)**	**−0.6 (0.1)**	**−1.0 (0.1)**	**−1.2 (0.1)**	0.17	<0.001	<0.001	0.003	<0.001	0.14
Energy Absorption at Knee (J/kg)										
Non-op	1.7 (0.1)	1.8 (0.1)	2.3 (0.1)	2.3 (0.1)	0.61	0.01	0.03	0.004	0.003	0.95
Op	1.3 (0.1)	1.3 (0.1)	1.1 (0.1)	1.0 (0.1)	0.93	0.33	0.27	0.20	0.09	0.68
Asymmetry	**−0.4 (0.1)**	**−0.6 (0.1)**	**−1.2 (0.1)**	**−1.2 (0.1)**	0.46	<0.001	<0.001	<0.001	<0.001	0.72

Results are presented as model predicted mean (SE) adjusting for sex, age, and time since surgery. Bold indicates significant difference between sides (asymmetry) within each group at *p* < 0.05. IT: iliotibial band; HT: hamstring tendon; QT: quadriceps tendon; PT: patellar tendon.

**Table A2:** Comparison of sagittal knee kinematics/kinetics for operative and non-operative limbs during cutting for patients with isolated ACLR (no concomitant meniscus procedure).

Cut	Mean (SE)				*p*-Value					
Isolated ACLR	IT (*n* = 17 Visits)	HT (*n* = 25 Visits)	QT (*n* = 24 Visits)	PT (*n* = 47 Visits)	HT vs. IT	QT vs. IT	PT vs. IT	QT vs. HT	PT vs. HT	PT vs. QT
Max Knee Angle (°)										
Non-op	63 (3)	67 (2)	70 (2)	68 (2)	0.21	0.07	0.33	0.41	0.84	0.27
Op	59 (3)	64 (2)	59 (2)	57 (2)	0.16	0.90	0.67	0.06	0.005	0.32
Asymmetry	−4 (2)	−3 (2)	**−11 (2)**	**−11 (1)**	0.81	0.005	0.003	0.001	<0.001	0.89
Max Knee Moment (N·m/kg)										
Non-op	2.6 (0.1)	2.8 (0.1)	3.2 (0.1)	3.1 (0.1)	0.27	0.003	0.02	0.006	0.03	0.51
Op	2.2 (0.1)	2.3 (0.1)	2.0 (0.1)	1.8 (0.1)	0.52	0.89	0.36	0.26	0.01	0.16
Asymmetry	**−0.4 (0.1)**	**−0.5 (0.1)**	**−1.2 (0.1)**	**−1.3 (0.1)**	0.55	<0.001	<0.001	<0.001	<0.001	0.43
Energy Absorption at Knee (J/kg)										
Non-op	0.9 (0.1)	1.0 (0.1)	1.3 (0.1)	1.2 (0.1)	0.32	0.003	0.03	0.004	0.03	0.38
Op	0.7 (0.1)	0.7 (0.1)	0.7 (0.1)	0.4 (0.1)	0.39	0.51	0.35	0.78	0.006	0.009
Asymmetry	**−0.2 (0.1)**	**−0.3 (0.1)**	**−0.6 (0.1)**	**−0.8 (0.1)**	0.89	0.007	<0.001	0.005	<0.001	0.10

Results are presented as model predicted mean (SE) adjusting for sex, age, and time since surgery. Bold indicates significant difference between sides (asymmetry) within each group at *p* < 0.05. IT: iliotibial band; HT: hamstring tendon; QT: quadriceps tendon; PT: patellar tendon.

**Table A3:** Comparison of sagittal knee kinematics/kinetics for operative and non-operative limbs during vertical drop jump for patients with concomitant meniscus repair, debridement, or meniscectomy.

Drop Jump	Mean (SE)				*p*-Value					
Concomitant Meniscus Procedure	IT (*n* = 7 Visits)	HT (*n* = 13 Visits)	QT (*n* = 24 Visits)	PT (*n* = 18 Visits)	HT vs. IT	QT vs. IT	PT vs. IT	QT vs. HT	PT vs. HT	PT vs. QT
Max Knee Angle (°)										
Non-op	118 (6)	109 (5)	113 (4)	120 (4)	0.23	0.56	0.87	0.43	0.25	0.54
Op	113 (6)	107 (5)	108 (4)	116 (4)	0.31	0.46	0.84	0.76	0.38	0.45
Asymmetry	−4 (3)	−3 (3)	**−6 (2)**	**−5 (2)**	0.71	0.71	0.90	0.33	0.52	0.74
Max Knee Moment (N·m/kg)										
Non-op	2.1 (0.2)	2.4 (0.1)	2.4 (0.1)	2.4 (0.1)	0.40	0.56	0.94	0.73	0.36	0.46
Op	1.7 (0.2)	1.4 (0.1)	1.3 (0.1)	1.4 (0.1)	0.10	0.01	0.04	0.24	0.44	0.73
Asymmetry	**−0.5 (0.2)**	**−1.0 (0.2)**	**−1.2 (0.1)**	**−1.0 (0.1)**	0.06	0.01	0.06	0.54	0.91	0.42
Energy Absorption at Knee (J/kg)										
Non-op	2.1 (0.2)	2.1 (0.1)	2.2 (0.1)	2.4 (0.1)	0.65	0.84	0.94	0.73	0.52	0.67
Op	1.3 (0.2)	1.2 (0.1)	1.1 (0.1)	1.2 (0.1)	0.39	0.13	0.33	0.32	0.74	0.51
Asymmetry	**−0.9 (0.2)**	**−0.9 (0.2)**	**−1.2 (0.1)**	**−1.1 (0.1)**	0.73	0.19	0.26	0.25	0.36	0.84

Results are presented as model predicted mean (SE) adjusting for sex, age, and time since surgery. Bold indicates significant difference between sides (asymmetry) within each group at *p* < 0.05. IT: iliotibial band; HT: hamstring tendon; QT: quadriceps tendon; PT: patellar tendon.

**Table A4:** Comparison of sagittal knee kinematics/kinetics for operative and non-operative limbs during cutting for patients with concomitant meniscus repair, debridement, or meniscectomy.

Cut	Mean (SE)				*p*-Value					
Concomitant Meniscus Procedure	IT (*n* = 7 Visits)	HT (*n* = 13 Visits)	QT (*n* = 24 Visits)	PT (*n* = 18 Visits)	HT vs. IT	QT vs. IT	PT vs. IT	QT vs. HT	PT vs. HT	PT vs. QT
Max Knee Angle (°)										
Non-op	68 (4)	64 (3)	69 (3)	66 (3)	0.33	0.80	0.54	0.09	0.79	0.14
Op	65 (4)	57 (3)	59 (3)	59 (3)	0.09	0.37	0.21	0.32	0.87	0.39
Asymmetry	−3 (4)	**−7 (3)**	**−10 (2)**	**−7 (2)**	0.40	0.11	0.32	0.40	0.89	0.44
Max Knee Moment (N·m/kg)										
Non-op	3.0 (0.2)	3.2 (0.2)	2.9 (0.1)	3.2 (0.1)	0.36	0.99	0.49	0.21	0.90	0.23
Op	2.4 (0.2)	2.0 (0.2)	1.9 (0.1)	2.1 (0.1)	0.13	0.10	0.37	0.84	0.57	0.36
Asymmetry	−0.5 (0.3)	**−1.3 (0.2)**	**−1.0 (0.2)**	**−1.1 (0.2)**	0.04	0.12	0.09	0.37	0.52	0.81
Energy Absorption at Knee (J/kg)										
Non-op	1.3 (0.2)	1.2 (0.1)	1.1 (0.1)	1.1 (0.1)	0.70	0.53	0.37	0.72	0.42	0.54
Op	1.0 (0.2)	0.6 (0.1)	0.6 (0.1)	0.6 (0.1)	0.04	0.07	0.10	0.82	0.90	0.92
Asymmetry	−0.3 (0.2)	**−0.7 (0.1)**	**−0.5 (0.1)**	**−0.5 (0.1)**	0.10	0.18	0.36	0.57	0.32	0.60

Results are presented as model predicted mean (SE) adjusting for sex, age, and time since surgery. Bold indicates significant difference between sides (asymmetry) within each group at *p* < 0.05. IT: iliotibial band; HT: hamstring tendon; QT: quadriceps tendon; PT: patellar tendon.

**Table A5:** Comparison of sagittal knee kinematics/kinetics for operative and non-operative limbs during vertical drop jump for patients under 16 years of age and visits at least 6 months post-surgery.

Drop Jump	Mean (SE)				*p*-Value					
Age < 16 yr and ≥6 Months Post-Surgery	IT (*n* = 16 Visits)	HT (*n* = 19 Visits)	QT (*n* = 22 Visits)	PT (*n* = 21 Visits)	HT vs. IT	QT vs. IT	PT vs. IT	QT vs. HT	PT vs. HT	PT vs. QT
Max Knee Angle (°)										
Non-op	118 (4)	110 (4)	117 (3)	114 (4)	0.94	0.33	0.52	0.11	0.25	0.76
Op	116 (4)	107 (4)	112 (3)	109 (4)	0.84	0.52	0.68	0.20	0.34	0.82
Asymmetry	−2 (1)	**−3 (1)**	**−5 (1)**	**−4 (1)**	0.59	0.18	0.26	0.40	0.55	0.81
Max Knee Moment (N·m/kg)										
Non-op	1.9 (0.1)	2.1 (0.1)	2.3 (0.1)	2.3 (0.1)	0.10	0.001	0.003	0.04	0.03	0.79
Op	1.6 (0.1)	1.5 (0.1)	1.3 (0.1)	1.3 (0.1)	0.81	0.15	0.20	0.10	0.12	1.00
Asymmetry	**−0.3 (0.1)**	**−0.6 (0.1)**	**−1.0 (0.1)**	**−1.0 (0.1)**	0.08	<0.001	<0.001	0.006	0.003	0.84
Energy Absorption at Knee (J/kg)										
Non-op	1.8 (0.1)	1.8 (0.1)	2.1 (0.1)	2.1 (0.1)	0.83	0.05	0.09	0.02	0.03	0.98
Op	1.4 (0.1)	1.3 (0.1)	1.1 (0.1)	1.1 (0.1)	0.74	0.33	0.40	0.38	0.42	0.99
Asymmetry	**−0.5 (0.1)**	**−0.6 (0.1)**	**−1.0 (0.1)**	**−1.0 (0.1)**	0.52	0.001	0.001	0.004	0.004	0.98

Results are presented as model predicted mean (SE) adjusting for sex, age, and time since surgery. Bold indicates significant difference between sides (asymmetry) within each group at *p* < 0.05. IT: iliotibial band; HT: hamstring tendon; QT: quadriceps tendon; PT: patellar tendon.

**Table A6:** Comparison of sagittal knee kinematics/kinetics for operative and non-operative limbs during cutting for patients under 16 years of age and visits at least 6 months post-surgery.

Cut	Mean (SE)				*p*-Value					
Age <16 yr and ≥6 Months Post-Surgery	IT (*n* = 16 Visits)	HT (*n* = 19 Visits)	QT (*n* = 22 Visits)	PT (*n* = 21 Visits)	HT vs. IT	QT vs. IT	PT vs. IT	QT vs. HT	PT vs. HT	PT vs. QT
Max Knee Angle (°)										
Non-op	68 (3)	66 (2)	72 (2)	67 (2)	0.61	0.49	0.51	0.07	0.71	0.03
Op	65 (3)	65 (2)	62 (2)	58 (2)	0.74	0.29	0.08	0.29	0.02	0.16
Asymmetry	−3 (2)	−2 (2)	**−11 (2)**	**−8 (2)**	0.73	0.001	0.01	<0.001	0.003	0.30
Max Knee Moment (N·m/kg)										
Non-op	2.8 (0.1)	2.8 (0.1)	3.2 (0.1)	2.9 (0.1)	0.81	0.04	0.43	0.01	0.36	0.13
Op	2.4 (0.1)	2.1 (0.1)	2.0 (0.1)	1.9 (0.1)	0.34	0.17	0.14	0.56	0.33	0.65
Asymmetry	**−0.4 (0.1)**	**−0.7 (0.1)**	**−1.2 (0.1)**	**−1.0 (0.1)**	0.12	<0.001	0.001	0.002	0.05	0.27
Energy Absorption at Knee (J/kg)										
Non-op	1.1 (0.1)	1.1 (0.1)	1.5 (0.1)	1.1 (0.1)	0.44	0.22	0.42	0.004	0.85	0.003
Op	0.9 (0.1)	0.7 (0.1)	0.7 (0.1)	0.5 (0.1)	0.17	0.11	0.006	0.76	0.02	0.03
Asymmetry	**−0.2 (0.1)**	**−0.3 (0.1)**	**−0.7 (0.1)**	**−0.6 (0.1)**	0.46	<0.001	0.004	0.003	0.03	0.42

Results are presented as model predicted mean (SE) adjusting for sex, age, and time since surgery. Bold indicates significant difference between sides (asymmetry) within each group at *p* < 0.05. IT: iliotibial band; HT: hamstring tendon; QT: quadriceps tendon; PT: patellar tendon.
